# Eyes-closed task-free electroencephalography in clinical trials for Alzheimer’s disease: an emerging method based upon brain dynamics

**DOI:** 10.1186/s13195-014-0086-x

**Published:** 2014-12-19

**Authors:** Elisabeth CW van Straaten, Philip Scheltens, Alida A Gouw, Cornelis J Stam

**Affiliations:** Department of Clinical Neurophysiology, VU University Medical Center, de Bolelaan 1118, P.O. box 7057, 1007 MB Amsterdam, The Netherlands; Nutricia Research, Nutricia Advanced Medical Nutrition, Utrecht Science Park, Uppsalalaan 12, 3584 CT Utrecht, The Netherlands; Alzheimer Center & Department of Neurology, VU University Medical Center, de Boelelaan 1118, P.O. box 7057, 1007 MB Amsterdam, the Netherlands

## Abstract

Electroencephalography (EEG) is a longstanding technique to measure electrical brain activity and thereby an indirect measure of synaptic activity. Synaptic dysfunction accompanies Alzheimer’s disease (AD) and EEG can be regarded as a potentially useful biomarker in this disease. Lately, emerging analysis techniques of time series have become available for EEG, such as functional connectivity and network analysis, which have increased the possibilities for use in AD clinical trials. In this review, we report the EEG changes in the course of AD, including slowing of the EEG oscillations, decreased functional connectivity in the higher-frequency bands, and decline in optimal functional network organization. We discuss the use of EEG in clinical trials and provide directions for future research.

## Review

### Introduction

Alzheimer’s disease (AD) is a degenerative disease characterized by pathological changes at the neuronal and synaptic level that eventually result in dementia at the subject level. The present therapeutic strategies have a modest and only symptomatic effect. It therefore remains essential to develop and test new interventional strategies.

Biomarkers can play a role as outcome measures in clinical trials. Classically, outcome measures in clinical trials in AD have focused on cognitive performance and behavioral outcome [1]. However, these outcome measures provide limited information on the mode of action of the pharmacological intervention, due to the lack of knowledge on the exact relationship between the neuronal level (where changes due to the intervention are assumed to occur) and the cognitive-functional level. Biomarkers can serve as a bridge between these two levels. Although several authors mention the need for suitable biomarkers, only few randomized clinical trials in AD have included investigations other than the cognitive and behavioral assessments [2].

Several investigational modalities are available that potentially can serve as biomarkers in AD clinical trials. Magnetic resonance imaging, functional magnetic resonance imaging (fMRI), cerebrospinal fluid analysis and positron emission tomography each assess different brain properties and are valuable for characterizing AD processes [2]. However, these methods do not take into account the high-frequency dynamics of brain activity, which is a hallmark property of communication between neurons and is expected to be impaired in AD [3].

Eyes-closed task-free (one of the so-called resting states) electroencephalography (EEG) measures oscillatory electrical brain activity and captures dynamical processes at the macroscopic scale. This technique has potential use in phase III trials because of its favorable investigational properties: it is relatively inexpensive, widely available, stable over time, and patient friendly. Additionally, recent developments, such as the application of network theory, increased the possibilities for EEG signal analysis of contributing to the understanding the mode of action of interventions in AD – in particular, in relation to (synaptic) connectivity.

This paper aims to reassess the potential role of eyes-closed task-free EEG in future AD clinical trials. We will report and discuss the results of EEG analyses in AD and in past clinical AD trials. We shall also review recent advances in EEG signal analysis and provide directions for their use in future trials.

### Introduction to electroencephalography in Alzheimer’s disease studies

EEG measures the fluctuating electrical field surrounding neurons. The EEG signal results from the sum of all transmembrane currents picked up by the EEG electrodes, where the most important contribution comes from synaptic activity. Other currents include fast action potentials, calcium spikes, intrinsic voltage-dependent currents, ligand-gate ion fluxes, gap junctions, neuron–glia interactions, and ephaptic effects [4]. Typically, the number of EEG electrodes that are placed on the scalp varies between 20 and 40, limiting the spatial resolution. On the other hand, EEG picks up the fluctuations in field potential with a very high temporal resolution (in the order of milliseconds).

AD is pathologically characterized by widespread neuronal cell loss and depositions of amyloid and tau aggregates, but previous studies have also suggested a link between AD and synaptic dysfunction [3]. The synaptic changes probably interfere with normal neuronal electrical activity, even at rest when no specific cognitive task is being performed, and eyes-closed task-free EEG picks up this resting-state activity.

Recent technical advances have increased the possibilities for EEG analysis. Digital EEG files usually have a relatively small size, which allows fast electronic exchange between recording centers; for example, for central analysis in multicenter trials. In addition, most parameter settings are adjustable to the analytical needs at any time after the recording. Furthermore, with increasing computational power, classic frequency analysis as well as advanced and complex studies of the EEG time series that add information on the organization of brain function can be performed.

### Electroencephalography frequency analysis

Computerized frequency analysis allows the objective quantification of the different frequency components of the complex digital EEG signal and is at the basis of many different types of advanced EEG analysis. This analysis has been widely applied in the context of AD.

EEG oscillations are often divided into several frequency bands (bins of adjacent frequencies) – such as delta band 0.5 to 4 Hz, theta band 4 to 8 Hz, alpha band 8 to 13 Hz (sometimes divided into alpha 1 band 8 to 10 Hz and alpha 2 band 10 to 13 Hz), beta band 13 to 30 Hz, and gamma band 30 to 70 Hz – and analyzed according to these bands. Care should be taken when interpreting the results of the gamma band, and to a lesser extent also the beta band, since EEG activity above 20 Hz, and especially activity above 30 Hz, is merely activity of the skull muscles rather than of the cerebral cortex [5].

The absolute and relative quantities of each frequency band within the total EEG signal are indicative of normal or pathological conditions and can be used to detect group differences. The dominant frequency, which is seen as a peak in the frequency spectrum, typically lies in the alpha frequency range in healthy awake adults. The analysis of these frequencies is one of the most widely available digital analysis techniques considering that EEG recording and reviewing systems are increasingly equipped with built-in tools for this purpose. In addition, frequency analysis is computationally fast and, since it is one of the most used analytical methods for EEG, results can be weighed against previous research. On the other hand, while the quantity of activity at a certain frequency measured at an EEG electrode is related to the amount of local synchronization of the underlying neuronal population, there is limited information on the phase relationship and synchronization between distant brain regions.

**Figure 1:**
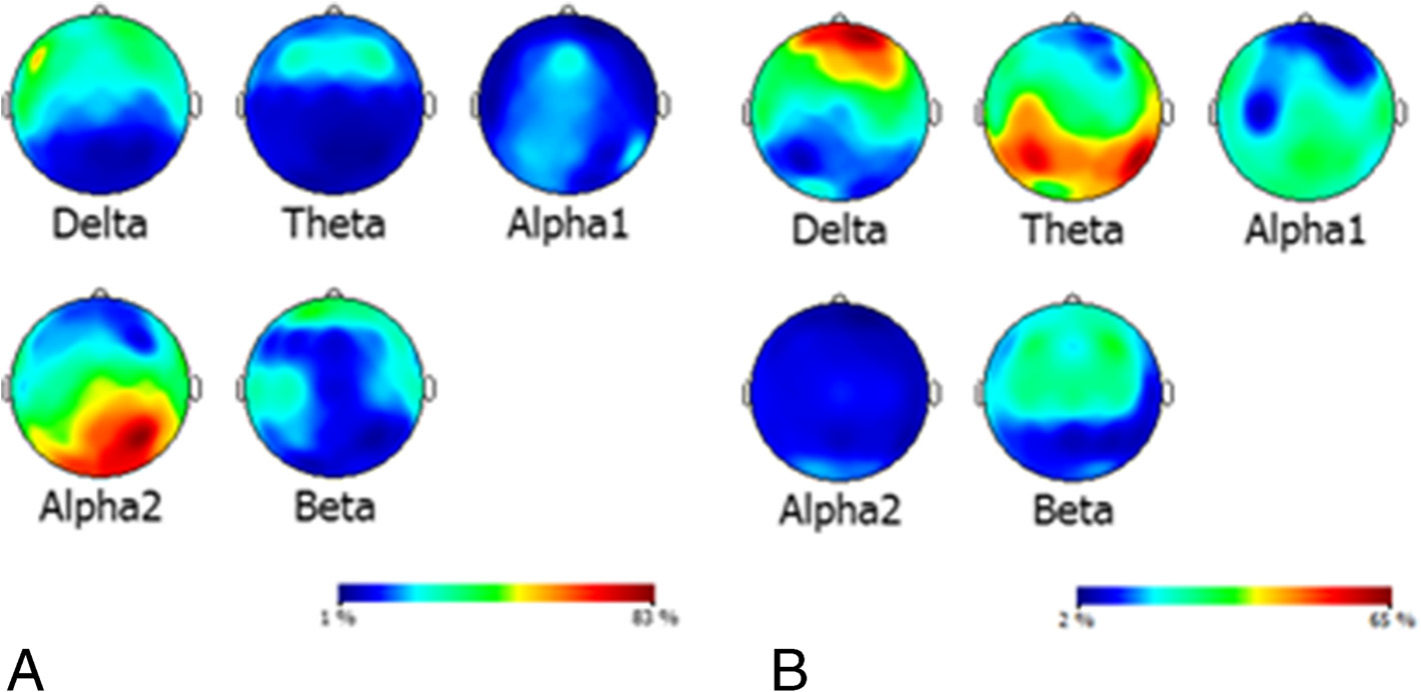
**Distribution of relative power across the brain.** Color-coded relative electroencephalography power maps for each frequency band of **(A)** a control subject (65 years old) and **(B)** an Alzheimer’s disease (AD) patient (66 years old, mild AD). Red, orange, and yellow, high relative power; blue and green, low relative power. The patient has a higher proportion of slow (delta and theta) activity, whereas the control has a higher proportion of faster (alpha) activity.

The most consistent EEG finding in AD is diffuse slowing of oscillations, displayed as an increased proportion of theta and delta activity and reduced peak frequency [6,7]. Longitudinal studies found progressive slowing during the course of the disease and also slowing in mild cognitive impairment at the group level to varying degrees [8,9]. Figure 1 shows an example of the difference in the distribution of relative power between a control subject and an AD patient. Cross-sectional studies reported EEG abnormalities depending on patient groups: abnormalities can be absent in late-onset AD and tend to be more prevalent in AD beginning at younger age [10,11]. Apolipoprotein E4 status has no unambiguous effect on EEG [12,13] and the EEG findings correlate with decreased psychometric performance in AD [14–16]. The mechanisms that cause the progressive EEG slowing in AD are not completely understood, but progressive loss of neuronal connectivity through synaptic dysfunction might play a role [17]. This view is supported by the findings that EEG slowing can be modeled as decreasing connectivity between computer-based neuronal assemblies [18] and that amyloid-beta toxicity primarily affects dendrites in AD [19]. On the other hand, other mechanisms that may influence connectivity between neurons, such as demyelination, might also be considered and may be the subject of future modeling studies.

### Electroencephalography in clinical trials

The slowing of EEG oscillations has been used as a marker for intervention effects in clinical trials, but the results have varied. Administration during several weeks to months of the approved AD treatments was not found to have a consistent effect on EEG. However, the trials were not double-blind controlled and therefore definite conclusions with respect to the properties of EEG as a marker in clinical trials cannot be drawn from these studies [20–28]. Two more recent studies from one double-blind controlled intervention trial in mild AD reported considerable information on trial design and procedures, which facilitates appreciation of the results [29,30]. These studies used frequency analysis (peak frequency and relative power), in addition to the more advanced functional connectivity and network analyses, as the EEG outcome parameter. The studies report that a 24-week intervention with medical food that was designed to enhance synaptic formation and function stabilized the peak frequency, whereas the control group declined on this measure. This indicates that, in untreated mild AD patients, a decline in peak frequency from baseline can be observed at a group level in the course of 6 months, and peak frequency is sensitive enough to pick up an intervention effect in this patient group.

The methodological differences and suboptimal trial designs of most intervention studies render the results difficult to interpret. Most studies are relatively old (before 2001) and do not meet the current standards on clinical trial design and reporting, such as a description of the randomization, the use of blinding, the description and statistical handling of dropouts, and the trial registration number. Furthermore, the number of participants is generally modest, with patient groups smaller than 20. These trial properties should prompt caution when interpreting and comparing the results.

Overall, the studies show that it is feasible to implement EEG in clinical trials and there are indications that peak frequency is sensitive to an intervention effect. Due to the insufficient quality of most clinical trial designs, most of the currently available EEG results are difficult to appreciate, but future studies that have implemented the present standards for clinical trials could resolve this issue.

### Functional connectivity

**Figure 2:**
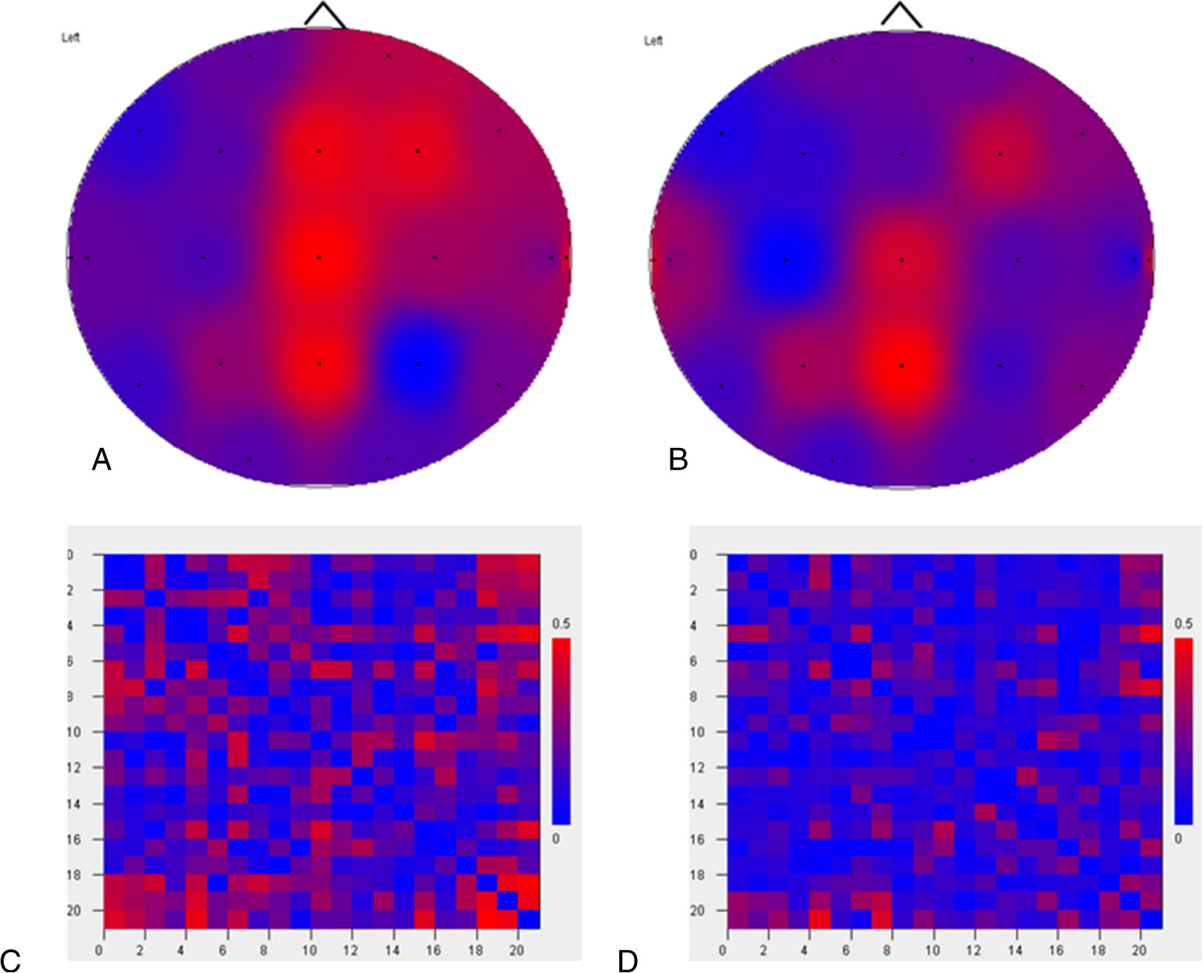
**Electroencephalography-based functional connectivity.** Functional connectivity (FC) is based on the phase lag index, filtered in the alpha 2 band (Fp1, Fp2, A1, and A2 excluded). **(A)** Distribution map for average FC of a healthy subject. **(B)** Distribution map of average FC for an Alzheimer’s disease patient. **(C)**, **(D)** Adjacency matrices of the control subject from (A) and the patient from (B), respectively, showing the strength of the pair-wise FC values. *X* and *Y* axes, electrode number; cells, color-coded FC value of the corresponding electrode pair. Red, high FC value; blue, low FC value. FC is generally lower in the patient. Pictures generated with BrainWave software version 0.9.117 [33].

Lately, the field of EEG analysis has advanced considerably and analysis methods beyond the classic frequency analysis have become available for AD research. One of these relatively new analyses is functional connectivity (see [31] for an overview of connectivity measures). The basis of functional connectivity lies in the notion that complex brain function, including cognition, requires large-scale integration of locally generated neural activity and that the synchronization of the activity between distributed brain areas is thought to reflect this integration [32]. Long-distance synchronization, as an approximation of functional connectedness, is quantified by the assessment of the statistical interdependency between time series (for which EEG next to fMRI and magnetoencephalography (MEG) is used) [31]. The resulting functional connectivity values indicate the strength of the functional coupling, based on the similarity of the signals (Figure 2). In addition to the most commonly used undirectional measures, effective connectivity measures exist that not only study the connectivity strength but also its direction, although care should be taken when interpreting the results with respect to the underlying biological dynamic processes.

The number of functional connectivity studies, including those that use EEG, in AD is rapidly increasing and AD-related connectivity changes are starting to become clear. Especially in the higher-frequency bands, AD patients have lower functional connectivity than controls [34,35]. Moreover, the connectivity changes are not equally distributed across brain regions and, at the regional level, connectivity increases as well as decreases have been described [36]. Positive relationships between decreased functional connectivity and impaired cognitive performance seem to be present [35,37]. Knyazeva and colleagues showed that the decrease of functional connectivity is related to the rate of cognitive decline in a 1-year follow-up study, but only in rapidly progressive patients and only in some specific brain areas that are known to be involved early in the AD process (especially the left-sided, lateral, and medial temporal regions) [38]. In addition, varying results were reported in mild cognitive impairment.

The application and interpretation of this new method requires knowledge about some methodological issues. One of these issues is the fact that the choice of the functional connectivity measure influences the results [39–41]. Ansari-Asl and colleagues showed in a model of coupled neural masses that the Pearson’s correlation coefficient was most sensitive to modeled connectivity, but in a model of a Rössler coupled system, for example, the synchronization likelihood was most sensitive [39]. David and colleagues reported that for broadband analysis the methods based on generalized synchronization are more sensitive than methods which use mutual information, but for narrow-band signals the opposite is true [40].

For intracranial EEG in rats, several connectivity measures – except for a mutual information measure – performed qualitatively comparably [41]. Additionally, each functional imaging method (EEG, MEG, fMRI) has modality-specific difficulties. For EEG, a major challenge is the ‘common-source problem’: when activity from one cortical source is projected to several surrounding electrodes, the electrodes have perfect zero-lag phase coupling. This common-source effect, or volume conduction, could lead to artificially high functional connectivity values for neighboring brain areas that do not reflect true functional coupling of separate neuronal systems. Zero-lag phase relations can be excluded using novel synchronization measures, but this, in turn, has the disadvantage of also excluding true zero-lag interactions [42,43]. EEG-derived functional connectivity is therefore either biased towards short-distance connections (when measures are used that include zero-lag phase relations) or towards longer-distance connections (when zero-lag relations are excluded). Other factors that influence connectivity results are the choice of the reference electrode and the choice of the length of the selected time series, both of which are not standardized in the field of functional connectivity research. Methodological differences between studies account for at least part of the difference in results and future studies are needed to further assess the relationship between methods.

In conclusion, the EEG-based functional connectivity in high-frequency bands is generally lower in AD than in controls. The significance of this finding lies in the support that it gives to the involvement of large-scale brain communication in this type of dementia and thereby to the involvement of synaptic activity. This is in line with the quantitative EEG analysis results regarding the concept of synaptic loss: the AD process is accompanied by a loss of synapses that induces a loss of long-distance functional connectivity which is picked up by EEG connectivity measures. However, other processes might also contribute to the reduction of EEG functional connectivity in AD; for example, by interfering with the timing of synaptic firing or other cortical processes that influence EEG signals. One of these processes might be demyelination, which has been demonstrated to be present in AD in the white matter underlying the cortex [44]. Future studies can further characterize the changes in functional connectivity in AD to increase the understanding of the effects of AD-related pathophysiological mechanisms on brain dynamics and thereby to decrease the gap between the neuronal level and the behavioral level of impaired cognition.

### Network analysis

Lately, graph theoretical analysis of complex networks has been implemented in brain research, including AD studies (for a review on brain networks, see [45]). Brain network analysis uses the analysis of functional connectivity by integrating the pair-wise functional correlation values into one network that can be characterized. In the case of EEG, network nodes are usually the brain regions underlying the electrodes and the functional connectivity value of each node pair (usually represented in a connectivity matrix) is used as a functional connection between nodes.

**Figure 3:**
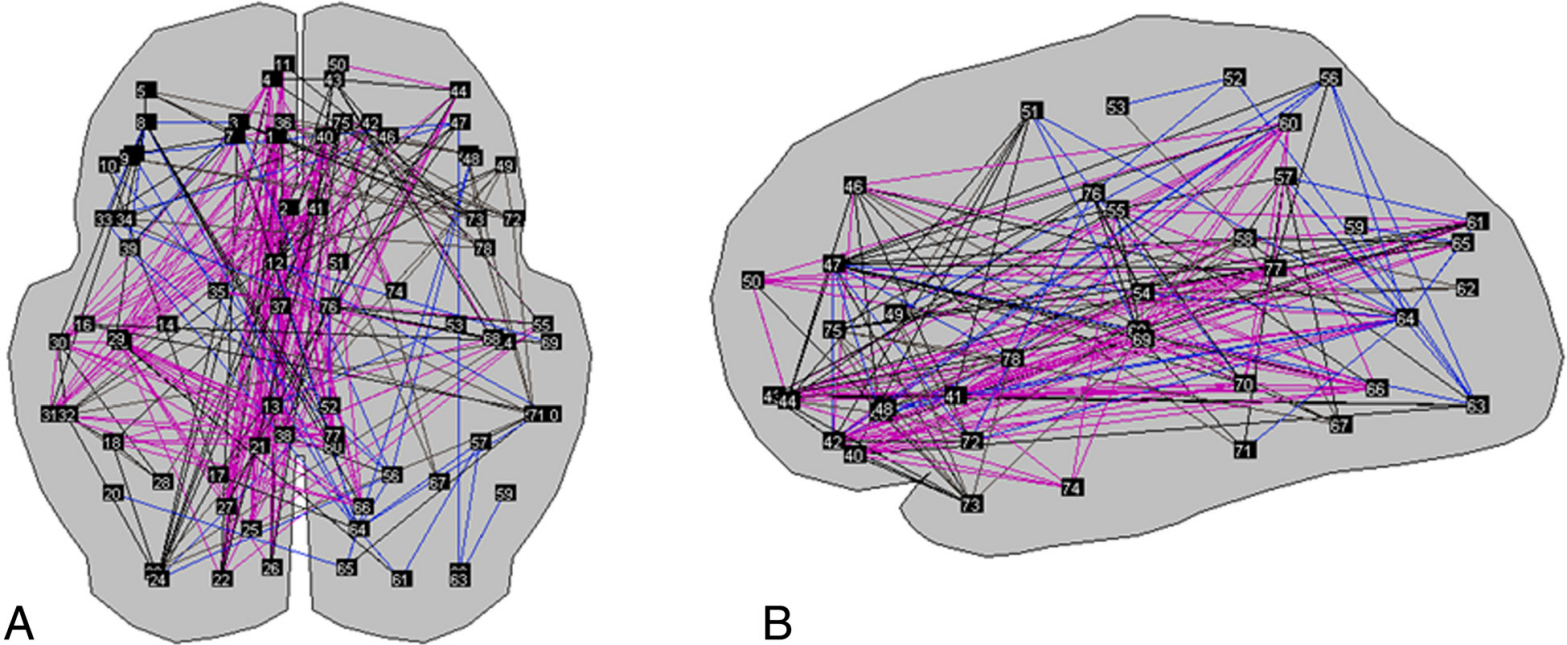
**Representation of a magnetoencephalography-based functional brain graph with 78 brain regions (network nodes indicated by numbers).** Connections (lines between nodes) are the thresholded functional connectivity values (phase lag index) between node pairs. **(A)** Transversal view, frontal up. **(B)** Sagittal view, frontal left. Colors indicate clusters of brain regions that have a higher connectivity within the cluster than outside the cluster (modules). A predominance of midline transversal connections can be seen. Pictures generated with BrainWave software version 0.9.117 [33].

As an example, Figure 3 shows a graphical representation of a network with 78 nodes, based on an MEG study from an AD patient. Network analysis quantifies higher-order patterns of the set of connections, such as the distribution of the connections across the nodes, and thereby uncovers information on the organizational properties of the functional network that extends beyond the functional connectivity analysis alone. This network perspective has revealed some aspects of the large-scale organization of cerebral activity: when regarded as a complex network of interconnected units (based on the functional connectivity values), functional brain networks fit the mathematical models for small-world (optimally combining short-distance connectivity and long-distance integration) and hierarchical modular organization (with inter-nested communities of highly interconnected nodes) better than the model of random networks [46–48].

The strengths of the network approach in AD studies include the implementation of a theoretical framework and powerful mathematical models to study the determinants of network disruption in AD. The mechanisms that change the macroscopic functional organization in AD might contribute to our understanding of the clinical signs as well as the pathophysiological processes in this disease. In addition, the theoretical framework of network analysis is invariant of the modality used and can be applied to any study that consists of measurement points with a relationship between these points. This facilitates the integration of results of brain mapping techniques, including time series (fMRI, EEG, MEG), structural studies (magnetic resonance imaging) and metabolic and perfusion studies (positron emission tomography, single-photon emission computerized tomography).

Indeed, the EEG-based functional brain networks of AD patients have been found to differ from those of healthy individuals. In one study, a change from the optimal small-world network configuration into a disorganized, more random network type was found in the higher-frequency bands, although volume conduction effects were included in this study [49]. In the lower-frequency bands this pattern is opposite, and both patterns have been found in the intermediate alpha 1 band [49,50]. AD seems to influence functional networks in a nonhomogeneous manner: the quantity of regional amyloid deposition has a linear relationship to the number of functional connections of that region [51]. The brain areas with a high level of functional connectivity (so-called hubs) and also a high amyloid load include the posterior cingulate and precuneus, the lateral temporal and parietal cortices, and parts of the prefrontal cortex. A computer model study showed that damaging the functional brain network as a function of the number of connections is more representative of the AD networks than random damage [18]. Additionally, another computer model suggested that the level of electrical activity, measured as spike density and total power, in a brain area increases with the number of connections in that area [52]. Taken together, the cross-sectional network analyses support the hypothesis that AD is a hub disease, with largest damage to the most central, well connected, and electrically most active brain regions resulting in disruption of the efficient small-world hierarchical modular network organization. One double-blind controlled intervention study used graph theoretical measures as secondary outcome measures [29]. In this study, functional brain networks of a group of mild AD patients receiving medical food that aimed to enhance synaptic formation and function were stable during 6 months, whereas the networks of the control group seemed to change in the direction of more randomness (with decreasing values for the clustering coefficient as a measure of local connectivity and for the path length as a measure of global integration).

Although a general pattern of AD-related network changes is emerging from the literature, methodological differences between studies influence the results, reducing the comparability across studies [53,54]. Functional brain networks are derived according to different strategies. One of the most straightforward networks is the unweighted network: connections are defined by setting a threshold, and only the connectivity values that exceed the threshold are included in the network. As a result, the network matrix is binary (the connections are yes/no present) and all connections have equal strength. The handling and visualization of these unweighted networks is relatively easy. On the other hand, determining the threshold value is arbitrary and the network values are heavily influenced by the functional connectivity values. A high threshold results in networks with fewer connections than a lower threshold and results in network values that indicate a decrease in local connectivity and global integration. In addition, applying an equal threshold to networks of different groups of subjects could result in unconnected nodes and differences in network size. Size differences hamper comparison between networks.

An alternative approach is the construction of weighted networks, in which the exact values of the connectivity matrix are preserved without the need for thresholding. Nevertheless, functional connectivity also influences weighted network measures to a great extent. A partial solution to this problem is normalizing the weighted networks. This can be done by presenting the network measures as proportions of measures of a set of random networks with equal connectivity strength, derived from shuffling the functional connectivity matrix. However, it has become clear that the resulting random networks are not really random and still contain a great deal of structure. In addition, the methodological differences in the computation of functional connectivity also influence network results, since the functional connectivity values are used for the computation of functional networks. The use of methods that do not require arbitrary parameter settings but uniquely follow from the connectivity matrix can resolve some of these problems.

**Figure 4:**
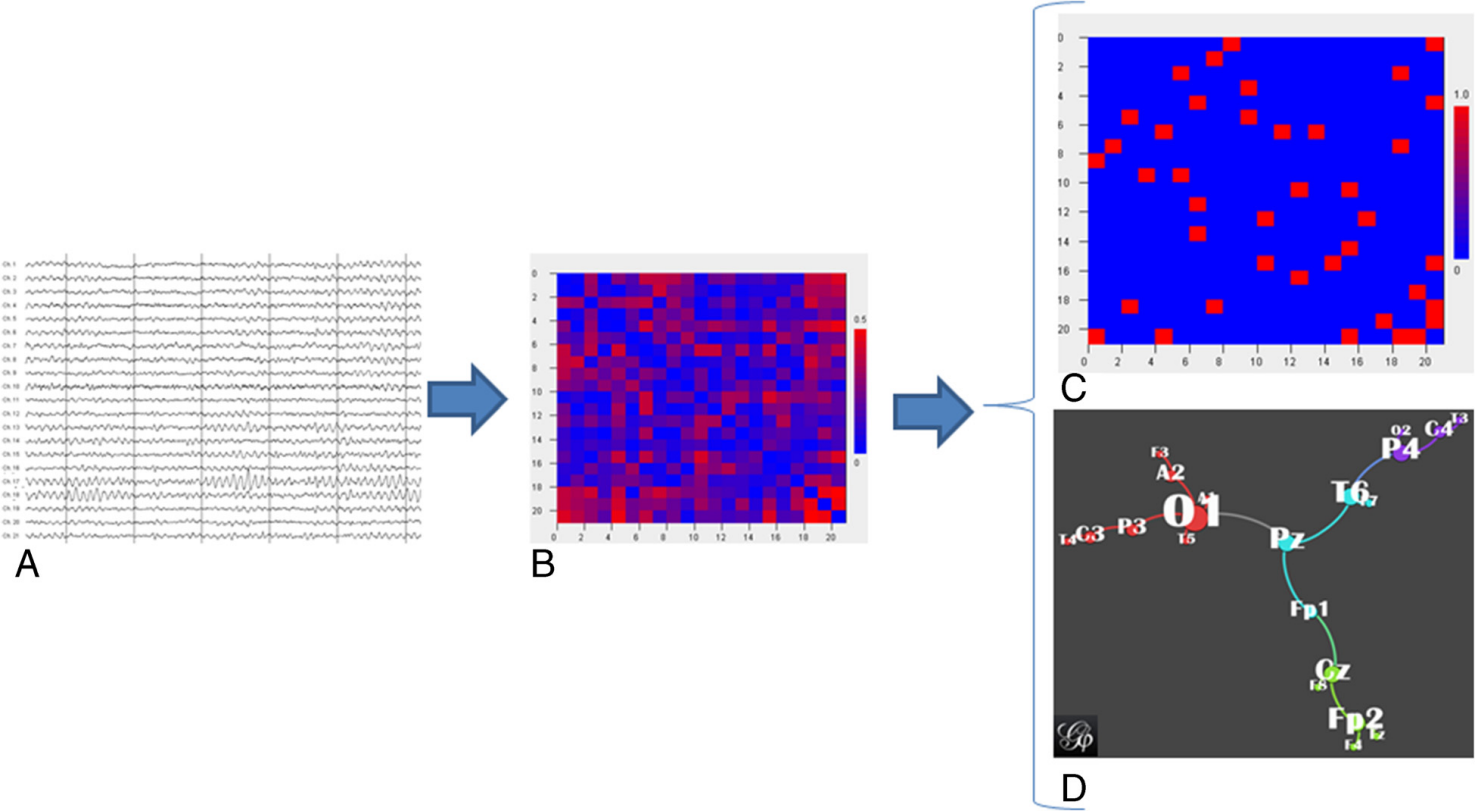
**Example of an electroencephalography-based minimum spanning tree of a healthy subject. (A)** Part of one of the four electroencephalography epochs used for the analysis. Every line is the time series of one electrode. **(B)** Phase lag index-based functional connectivity adjacency matrix with the functional connectivity values of each electrode pair. **(C)** Minimum spanning tree matrix derived from the matrix in **(B)** showing only the highest 20 connectivity values of the network without loops. **(D)** Graphic representation of **(C)**, colors indicate different network modules. Pictures generated with BrainWave software version 0.9.117 [33] **(A, B, C)** or Gephi version 0.8.2 **(D)** [59].

One such approach is the minimum spanning tree [55]. Figure 4 shows the workflow for constructing a minimum spanning tree. Here, four EEG epochs of 8.2 seconds for a healthy subject were used as input and the functional connectivity matrix for this subject was computed. Instead of taking the full matrix, only the connections with the highest values are considered for further network analysis in such a way that all nodes are connected and no loops are formed. While this method is unbiased, the minimum spanning tree consists of a relatively low number of connections and may under-represent less strong, but nonetheless important, connections. Also, the loopless structure of the network is not typical for the underlying microscopic brain connectivity, which consists of numerous feedback and feedforward loops, making this network somewhat more indirect. One fMRI study showed that the organization of such networks is disturbed in AD [56]. Whether minimum spanning tree changes in EEG in AD can be found remains to be studied, but changes were found over the course of 2 years in healthy children and after sleep deprivation in children with focal epilepsy [57,58].

Although a longitudinal MEG study found network changes to be correlated with the decline in cognitive performance in Parkinson’s disease, the longitudinal behavior of network changes in AD has not so far been studied. It can be conceived that the changes do not relate linearly to disease progression, as is the case for other modalities such as hippocampal atrophy and memory scores, and that the yield of EEG network measures in clinical trials for AD depends on the AD severity. To what extent network measures suffer from floor or ceiling effects, which could make them less sensitive in earlier or later disease stages respectively, is currently insufficiently known. Longitudinal studies, as well as measuring an intervention effect in different AD stages, will help to establish the value of this type of marker.

## Conclusions

This review presents an overview of eyes-closed task-free EEG analysis methods that are potentially useful in AD clinical trials and discusses past intervention studies implementing EEG in AD. Studies using conventional methods show a converging pattern of change in AD. This pattern of slowing of EEG oscillatory activity may serve as a benchmark in intervention studies. In addition, newer analysis methods, such as functional connectivity and network analysis, have been applied to EEG in AD and, when implemented in clinical trials, can contribute to the understanding the mode of action of the interventions. For example, an intervention-related preservation of local connectivity that is distributed across the brain regions according to the hub distribution can indicate that the intervention is especially effective in hub regions and thereby in the brain regions with the highest electrical activity and the highest metabolic demand. In addition, different intervention effects on long-distance and short-distance connectivity indicate what systems might be involved. However, it is presently too early to apply functional connectivity and network analyses for these hypotheses and more knowledge on the behavior of the network and connectivity measures is needed to fully understand their meaning.

The value of EEG in clinical trials will improve when it is implemented in high-quality randomized clinical trials. So far, most controlled trials using EEG are very small and do not report enough details on the trial design and trial execution to assess the risk of bias. Furthermore, most studies are relatively old and not designed according to the current standards. However, with advances in designing and reporting of randomized controlled trials, future studies can address these problems. We recommend that the quality standards which apply to the performance of clinical trials should also be implemented in the EEG part of future studies.

In addition, knowledge of the influence of the type of EEG analysis can improve comparison of results between studies. No universal method exists at each analysis level (quantitative analysis, connectivity analysis, and network analysis) and each method influences the outcome. Consequently, studies that use different analysis techniques are difficult to compare. When test characteristics are taken into account, however, differences in results might explain differences between studies and might even give additional information on the mode of action. The strategy of using multiple measures and searching for a consistent direction of connectivity changes in AD across the methods has the advantage of increasing knowledge on the behavior, such as sensitivity, specificity, availability, and applicability, of the different measures relative to each other. An alternative approach would be to further subdivide functional connectivity into categories based on the characteristics of the synchronization measures, such as a subdivision into short-distance and long-distance biased connectivity. In this way, it may be possible to interpret the results of different connectivity measures in addition to, rather than instead of, each other. In parallel, there is a need for the development of EEG analysis techniques that do not depend upon the often arbitrary choice of one or more parameters. The availability of an EEG analysis method that has good discriminative properties and which renders results without a single choice in parameter settings could be valuable for reaching consensus.

At this moment, clinical measures are the only accepted endpoints for pivotal phase III intervention studies in AD [60]. It is also known that clinical signs do not linearly relate to cell loss or the characteristic AD pathology of tangles and plaques. Cellular changes precede cognitive disturbance, but the exact temporal relationship remains unclear. One study has reported a relationship between cellular loss in the entorhinal cortex and loss of cognitive performance prior to, more than during, AD [61]. Additionally, the pathological AD features vary over the cortical regions, and the quantification of AD pathology is therefore also variable and dependent on the regions of interest and sampling. At the other end, performance on cognitive testing is also subject to other factors than the AD process. Typically, performance varies in time to a certain extent due to fluctuations in concentration, learning effects, and other intrinsic and extrinsic factors. There are therefore several arguments as to why looking only at clinical performance is not sufficient to understand an intervention effect in an AD trial. Coordinated brain activity, such as measured by EEG, and its large-scale organization are becoming increasingly identified as meaningful brain characteristics and possible candidates for the assessment of interventions in AD [62].

In conclusion, EEG in clinical trials in AD is a feasible and potentially useful addition to cognitive endpoint measures in AD trials. Recent developments in EEG signal analysis, such as network analysis, present promising methods to provide frameworks for the testing of hypotheses on the mode of action of interventions. When the quality of the clinical trial design and analysis meets the current standards, the role of EEG in clinical trials for AD can be fully appreciated.

## Abbreviations

AD: Alzheimer’s disease
EEG: Electroencephalography
fMRI: Functional magnetic resonance imaging
MEG: Magnetoencephalography

## References

[1] Berk C, Sabbagh MN (2013). Successes and failures for drugs in late-stage development for Alzheimer’s disease. Drugs Aging.

[2] Hampel H, Wilcock G, Andrieu S, Aisen P, Blennow K, Broich K, Carrillo M, Fox NC, Frisoni GB, Isaac M, Lovestone S, Nordberg A, Prvulovic D, Sampaio C, Scheltens P, Weiner M, Winblad B, Coley N, Vellas B, Oxford Task Force Group (2011). Biomarkers for Alzheimer’s disease therapeutic trials. Prog Neurobiol.

[3] Selkoe DJ (2002). Alzheimer’s disease is a synaptic failure. Science (NY).

[4] Buzsaki G, Anastassiou CA, Koch C (2012). The origin of extracellular fields and currents – EEG, ECoG, LFP and spikes. Nat Rev Neurosci.

[5] Whitham EM, Pope KJ, Fitzgibbon SP, Lewis T, Clark CR, Loveless S, Broberg M, Wallace A, DeLosAngeles D, Lillie P, Hardy A, Fronsko R, Pulbrook A, Willoughby JO (2007). Scalp electrical recording during paralysis: quantitative evidence that EEG frequencies above 20 Hz are contaminated by EMG. Clin Neurophysiol.

[6] Jeong J (2004). EEG dynamics in patients with Alzheimer’s disease. Clin Neurophysiol.

[7] Jackson CE, Snyder PJ (2008). Electroencephalography and event-related potentials as biomarkers of mild cognitive impairment and mild Alzheimer’s disease. Alzheimers Dement.

[8] Babiloni C, Del Percio C, Lizio R, Marzano N, Infarinato F, Soricelli A, Salvatore E, Ferri R, Bonforte C, Tedeschi G, Montella P, Baglieri A, Rodriguez G, Famà F, Nobili F, Vernieri F, Ursini F, Mundi C, Frisoni GB, Rossini PM (2014). Cortical sources of resting state electroencephalographic alpha rhythms deteriorate across time in subjects with amnesic mild cognitive impairment. Neurobiol Aging.

[9] Forstl H, Sattel H, Besthorn C, Daniel S, Geiger-Kabisch C, Hentschel F, Sarochan M, Zerfass R (1996). Longitudinal cognitive, electroencephalographic and morphological brain changes in ageing and Alzheimer’s disease. Br J Psychiatry.

[10] de Waal H, Stam CJ, de Haan W, van Straaten EC, Scheltens P, van der Flier WM (2012). Young Alzheimer patients show distinct regional changes of oscillatory brain dynamics. Neurobiol Aging.

[11] Klass DW, Brenner RP (1995). Electroencephalography of the elderly. J Clin Neurophysiol.

[12] Ponomareva NV, Korovaitseva GI, Rogaev EI (2008). EEG alterations in non-demented individuals related to apolipoprotein E genotype and to risk of Alzheimer disease. Neurobiol Aging.

[13] de Waal H, Stam CJ, de Haan W, van Straaten EC, Blankenstein MA, Scheltens P, van der Flier WM (2013). Alzheimer’s disease patients not carrying the apolipoprotein E epsilon4 allele show more severe slowing of oscillatory brain activity. Neurobiol Aging.

[14] Saletu B, Anderer P, Paulus E, Grunberger J, Wicke L, Neuhold A, Fischhof PK, Litschauer G (1991). EEG brain mapping in diagnostic and therapeutic assessment of dementia. Alzheimer Dis Assoc Disord.

[15] Muller TJ, Thome J, Chiaramonti R, Dierks T, Maurer K, Fallgatter AJ, Frolich L, Scheubeck M, Strik WK (1997). A comparison of qEEG and HMPAO-SPECT in relation to the clinical severity of Alzheimer’s disease. Eur Arch Psychiatry Clin Neurosci.

[16] Gianotti LR, Kunig G, Lehmann D, Faber PL, Pascual-Marqui RD, Kochi K, Schreiter-Gasser U (2007). Correlation between disease severity and brain electric LORETA tomography in Alzheimer’s disease. Clin Neurophysiol.

[17] Abuhassan K, Coyle D, Maguire LP (2012). Investigating the neural correlates of pathological cortical networks in Alzheimer’s disease using heterogeneous neuronal models. IEEE Trans BioMed Eng.

[18] Stam CJ, de Haan W, Daffertshofer A, Jones BF, Manshanden I, van Cappellen van Walsum AM, Montez T, Verbunt JP, de Munck JC, van Dijk BW, Berendse HW, Scheltens P (2009). Graph theoretical analysis of magnetoencephalographic functional connectivity in Alzheimer’s disease. Brain.

[19] Thal DR, Griffin WS, Braak H (2008). Parenchymal and vascular Aβ-deposition and its effects on the degeneration of neurons and cognition in Alzheimer’s disease. J Cell Mol Med.

[20] Adler G, Brassen S (2001). Short-term rivastigmine treatment reduces EEG slow-wave power in Alzheimer patients. Neuropsychobiology.

[21] Adler G, Brassen S, Chwalek K, Dieter B, Teufel M (2004). Prediction of treatment response to rivastigmine in Alzheimer’s dementia. J Neurol Neurosurg Psychiatry.

[22] Balkan S, Yaras N, Mihci E, Dora B, Agar A, Yargicoglu P (2003). Effect of donepezil on EEG spectral analysis in Alzheimer’s disease. Acta Neurol Belg.

[23] Brassen S, Adler G (2003). Short-term effects of acetylcholinesterase inhibitor treatment on EEG and memory performance in Alzheimer patients: an open, controlled trial. Pharmacopsychiatry.

[24] Gianotti LR, Kunig G, Faber PL, Lehmann D, Pascual-Marqui RD, Kochi K, Schreiter-Gasser U (2008). Rivastigmine effects on EEG spectra and three-dimensional LORETA functional imaging in Alzheimer’s disease. Psychopharmacology (Berl).

[25] Kogan EA, Korczyn AD, Virchovsky RG, Klimovizky S, Treves TA, Neufeld MY (2001). EEG changes during long-term treatment with donepezil in Alzheimer’s disease patients. J Neural Transm.

[26] Reeves RR, Struve FA, Patrick G (2002). The effects of donepezil on quantitative EEG in patients with Alzheimer’s disease. Clin Electroencephalogr.

[27] Rodriguez G, Vitali P, Canfora M, Calvini P, Girtler N, De Leo C, Piccardo A, Nobili F (2004). Quantitative EEG and perfusional single photon emission computed tomography correlation during long-term donepezil therapy in Alzheimer’s disease. Clin Neurophysiol.

[28] Rodriguez G, Vitali P, De Leo C, De Carli F, Girtler N, Nobili F (2002). Quantitative EEG changes in Alzheimer patients during long-term donepezil therapy. Neuropsychobiology.

[29] de Waal H, Stam CJ, Lansbergen MM, Wieggers RL, Kamphuis PJ, Scheltens P, Maestu F, van Straaten EC (2014). The effect of souvenaid on functional brain network organisation in patients with mild Alzheimer’s disease: a randomised controlled study. PLoS One.

[30] Scheltens P, Twisk JW, Blesa R, Scarpini E, von Arnim CA, Bongers A, Harrison J, Swinkels SH, Stam CJ, de Waal H, Wurtman RJ, Wieggers RL, Vellas B, Kamphuis PJ (2012). Efficacy of Souvenaid in mild Alzheimer’s disease: results from a randomized, controlled trial. J Alzheimers Dis.

[31] Pereda E, Quiroga RQ, Bhattacharya J (2005). Nonlinear multivariate analysis of neurophysiological signals. Prog Neurobiol.

[32] Uhlhaas PJ, Pipa G, Lima B, Melloni L, Neuenschwander S, Nikolic D, Singer W (2009). Neural synchrony in cortical networks: history, concept and current status. Front Integr Neurosci.

[33] **Connected Brains** [http://home.kpn.nl/stam7883/]

[34] Koenig T, Prichep L, Dierks T, Hubl D, Wahlund LO, John ER, Jelic V (2005). Decreased EEG synchronization in Alzheimer’s disease and mild cognitive impairment. Neurobiol Aging.

[35] Park YM, Che HJ, Im CH, Jung HT, Bae SM, Lee SH (2008). Decreased EEG synchronization and its correlation with symptom severity in Alzheimer’s disease. Neurosci Res.

[36] Knyazeva MG, Jalili M, Brioschi A, Bourquin I, Fornari E, Hasler M, Meuli R, Maeder P, Ghika J (2010). Topography of EEG multivariate phase synchronization in early Alzheimer’s disease. Neurobiol Aging.

[37] Stam CJ, van der Made Y, Pijnenburg YA, Scheltens P (2003). EEG synchronization in mild cognitive impairment and Alzheimer’s disease. Acta Neurol Scand.

[38] Knyazeva MG, Carmeli C, Khadivi A, Ghika J, Meuli R, Frackowiak RS (2013). Evolution of source EEG synchronization in early Alzheimer’s disease. Neurobiol Aging.

[39] Ansari-Asl K, Senhadji L, Bellanger JJ, Wendling F (2006). Quantitative evaluation of linear and nonlinear methods characterizing interdependencies between brain signals. Phys Rev E Stat Nonlin Soft Matter Phys.

[40] David O, Cosmelli D, Friston KJ (2004). Evaluation of different measures of functional connectivity using a neural mass model. Neuroimage.

[41] Quian Quiroga R, Kraskov A, Kreuz T, Grassberger P (2002). Performance of different synchronization measures in real data: a case study on electroencephalographic signals. Phys Rev E Stat Nonlin Soft Matter Phys.

[42] Nolte G, Bai O, Wheaton L, Mari Z, Vorbach S, Hallett M (2004). Identifying true brain interaction from EEG data using the imaginary part of coherency. Clin Neurophysiol.

[43] Stam CJ, Nolte G, Daffertshofer A (2007). Phase lag index: assessment of functional connectivity from multi channel EEG and MEG with diminished bias from common sources. Hum Brain Mapp.

[44] Fornari E, Maeder P, Meuli R, Ghika J, Knyazeva MG (2012). Demyelination of superficial white matter in early Alzheimer’s disease: a magnetization transfer imaging study. Neurobiol Aging.

[45] Sporns O (2014). Contributions and challenges for network models in cognitive neuroscience. Nat Neurosci.

[46] Bullmore E, Sporns O (2009). Complex brain networks: graph theoretical analysis of structural and functional systems. Nat Rev Neurosci.

[47] Stam CJ, van Straaten EC (2012). The organization of physiological brain networks. Clin Neurophysiol.

[48] Watts DJ, Strogatz SH (1998). Collective dynamics of ‘small-world’ networks. Nature.

[49] de Haan W, Pijnenburg YA, Strijers RL, van der Made Y, van der Flier WM, Scheltens P, Stam CJ (2009). Functional neural network analysis in frontotemporal dementia and Alzheimer’s disease using EEG and graph theory. BMC Neurosci.

[50] Vecchio F, Miraglia F, Marra C, Quaranta D, Vita MG, Bramanti P, Rossini PM (2014). Human brain networks in cognitive decline: a graph theoretical analysis of cortical connectivity from EEG data. J Alzheimers Dis.

[51] Buckner RL, Sepulcre J, Talukdar T, Krienen FM, Liu H, Hedden T, Andrews-Hanna JR, Sperling RA, Johnson KA (2009). Cortical hubs revealed by intrinsic functional connectivity: mapping, assessment of stability, and relation to Alzheimer’s disease. J Neurosci.

[52] de Haan W, Mott K, van Straaten EC, Scheltens P, Stam CJ (2012). Activity dependent degeneration explains hub vulnerability in Alzheimer’s disease. PLoS Comput Biol.

[53] van Wijk BC, Stam CJ, Daffertshofer A (2010). Comparing brain networks of different size and connectivity density using graph theory. PLoS One.

[54] Tijms BM, Wink AM, de Haan W, van der Flier WM, Stam CJ, Scheltens P, Barkhof F (2013). Alzheimer’s disease: connecting findings from graph theoretical studies of brain networks. Neurobiol Aging.

[55] Stam CJ, Tewarie P, Van Dellen E, van Straaten EC, Hillebrand A, Van Mieghem P (2014). The trees and the forest: characterization of complex brain networks with minimum spanning trees. Int J Psychophysiol.

[56] Ciftci K (2011). Minimum spanning tree reflects the alterations of the default mode network during Alzheimer’s disease. Ann Biomed Eng.

[57] Boersma M, Smit DJ, Boomsma DI, De Geus EJ, de Waal HA D-v, Stam CJ (2013). Growing trees in child brains: graph theoretical analysis of electroencephalography-derived minimum spanning tree in 5- and 7-year-old children reflects brain maturation. Brain Connect.

[58] van Diessen E, Otte WM, Braun KP, Stam CJ, Jansen FE (2014). Does sleep deprivation alter functional EEG networks in children with focal epilepsy?. Front Syst Neurosci.

[59] **Gephi** [https://gephi.org/]

[60] Broich K, Weiergraber M, Hampel H (2011). Biomarkers in clinical trials for neurodegenerative diseases: regulatory perspectives and requirements. Prog Neurobiol.

[61] Price JL, Ko AI, Wade MJ, Tsou SK, McKeel DW, Morris JC (2001). Neuron number in the entorhinal cortex and CA1 in preclinical Alzheimer disease. Arch Neurol.

[62] Hampel H, Prvulovic D, Teipel S, Jessen F, Luckhaus C, Frolich L, Riepe MW, Dodel R, Leyhe T, Bertram L, Hoffmann W, Faltraco F, German Task Force on Alzheimer’s Disease (GTF-AD) (2011). The future of Alzheimer’s disease: the next 10 years. Prog Neurobiol.

